# Activation of GRP/GRP-R signaling contributes to castration-resistant prostate cancer progression

**DOI:** 10.18632/oncotarget.11326

**Published:** 2016-08-17

**Authors:** Jingbo Qiao, Magdalena M. Grabowska, Ingrid S. Forestier, Janni Mirosevich, Thomas C. Case, Dai H. Chung, Justin M.M. Cates, Robert J. Matusik, H. Charles Manning, Renjie Jin

**Affiliations:** ^1^ Department of Cancer Biology, Vanderbilt University Medical Center, Nashville, TN, USA; ^2^ Department of Pediatric Surgery, Vanderbilt University Medical Center, Nashville, TN, USA; ^3^ Vanderbilt Prostate Cancer Center and Department of Urologic Surgery, Vanderbilt University Medical Center, Nashville, TN, USA; ^4^ Department of Biochemistry, University of Puerto Rico, Medical Sciences Campus, San Juan, Puerto Rico; ^5^ Department of Pathology, Microbiology and Immunology, Vanderbilt University Medical Center, Nashville, TN, USA; ^6^ Institute of Imaging Science and Center for Molecular Probes, Vanderbilt University Medical Center, Nashville, TN, USA

**Keywords:** GRP/GRP-R, NF-kappa B, androgen receptor variants, prostate cancer, progression

## Abstract

Numerous studies indicate that androgen receptor splice variants (ARVs) play a critical role in the development of castration-resistant prostate cancer (CRPC), including the resistance to the new generation of inhibitors of androgen receptor (AR) action. Previously, we demonstrated that activation of NF-κB signaling increases ARVs expression in prostate cancer (PC) cells, thereby promoting progression to CRPC. However, it is unclear how NF-κB signaling is activated in CRPC. In this study, we report that long-term treatment with anti-androgens increases a neuroendocrine (NE) hormone — gastrin-releasing peptide (GRP) and its receptor (GRP-R) expression in PC cells. In addition, activation of GRP/GRP-R signaling increases ARVs expression through activating NF-κB signaling. This results in an androgen-dependent tumor progressing to a castrate resistant tumor. The knock-down of AR-V7 restores sensitivity to antiandrogens of PC cells over-expressing the GRP/GRP-R signaling pathway. These findings strongly indicate that the axis of Androgen-Deprivation Therapy (ADT) induces GRP/GRP-R activity, activation NF-κB and increased levels of AR-V7 expression resulting in progression to CRPC. Both prostate adenocarcinoma and small cell NE prostate cancer express GRP-R. Since the GRP-R is clinically targetable by analogue-based approach, this provides a novel therapeutic approach to treat advanced CRPC.

## INTRODUCTION

Numerous studies indicate that androgen receptor splice variants (ARVs) play a critical role in the development of castration-resistant prostate cancer (CRPC), including the resistance to the new generation of inhibitors of androgen receptor (AR) action [1–5]. ARVs, which lack the ligand-binding domain (LBD), are constitutively active in the absence of ligand [1–3]. Therefore, the traditional LBD targeted AR blockers cannot inhibit ARVs activation. The ARVs result in constitutive activation of the AR pathway thereby promoting prostate cancer (PC) cell growth at low concentrations of androgens [4, 6], enhance growth of androgen dependent xenografts in castrated mice [3] and the development of enzalutamide resistant PC [7]. Based on these findings, it has been proposed that ARVs can function as important drivers of CRPC [1–3, 5]. Therefore, understanding the mechanism responsible for ARVs expression in CRPC is critical to identify new targets for blocking the production of ARVs, thus treating CRPC. We and other researchers have shown that activation of NF-κB signaling increases ARVs expression in benign prostatic [8] and PC cells [9, 10] and blocking NF-κB signaling efficiently restores responsiveness of CRPC cells to anti-androgen treatment by decreasing ARVs expression [9]. Further, in metastatic PC, out of 104 pathways that are dysregulated, activation the NF-κB ranks in the top ten pathways [11] and we reported that a NF-κB gene signature predicts PC progression [12]. Although NF-κB signaling is a promising target in advanced CRPC, it has been difficult to develop drugs that block the oncogenic activity of NF-κB without interfering with its normal physiological roles, thus resulting in detrimental side effects. In addition, how NF-κB signaling is activated in response to Androgen-Deprivation Therapy (ADT) in PC patients is still not fully understood.

Neuroendocrine differentiation (NED) is becoming increasingly recognized as a mechanism that allows transdifferentiation of PC cells to escape ADT [13]. Previously, we showed that NED and increased neuroendocrine (NE) peptides, such as bombesin (BN) and gastrin-releasing peptide (GRP), contribute to CRPC through the activation of NF-κB and AR signaling [14, 15]. NE prostate cancer (NEPC; also known as small cell carcinoma) is rare at primary diagnosis [16–18], but NED (defined as prostatic adenocarcinoma that expresses NE markers) has long been recognized as a common occurrence in advanced PC [13, 19–22]. In the normal prostate, NE cells are rare and interspersed among the epithelium. These cells are believed to provide trophic signals to epithelial cell populations through the secretion of neuropeptides that can diffuse to influence surrounding cells. Although NE features are only detected in approximately 4% of primary PC [23–25], it is now estimated that at least 25% of patients with advanced PC eventually develop highly aggressive small cell NEPC [24, 26] and 40–100% of CRPC tumors acquire NED [23–25]. A new study has shown that 17/159 (10.7%) of CRPC patients have circulating tumor cells (CTCs) that are defined as NEPC since they have low or are absent for AR expression [21]. Further, transdifferentiation of CRPC adenocarcinomas to CRPC-NE tumors occurred in 30/81 (37%) of patients examined [13]. Thus, focal NED is increased within CRPC tumors as well as levels of NE-derived peptides such as GRP, neuron-specific enolase (NSE) and chromogranin-A in the serum of CRPC patients [13, 27–29]. It is now accepted that prostate adenocarcinoma cells have the capacity to undergo NE transdifferentiation (reviewed [30, 31]) as defined by the PC cells becoming NE-like due to the expression of NE markers [25, 30, 31]. Transdifferentiation of PC to an NE-like phenotype represents a noteworthy biological process that can be considered a consequence of the selective pressure induced by all treatments that lead to a fall in androgen levels, or to blocking the action of this steroid hormone [13, 25]. As transdifferentiation occurs, the CRPC adenocarcinoma undergoes a loss of AR expression to become an AR negative NEPC. This process occurs in stages where tumors with NE features may still have AR or express AR target genes. For example, Wang and Epstein examine 95 prostate primary small cell carcinomas and found that 25% were positive, often only focally, for prostate specific membrane antigen (PSMA) while 28% showed detectable prostate specific antigen (PSA) [32]. The development of NED in PC cells and the production of NE peptides, such as the NE hormone — GRP, by NE/NE-like cells are thought to be important mechanisms in the development of castration resistance [25].

GRP is a 27-amino acid neuropeptide that is the mammalian homologue of the linear tetradecapeptide BN originally isolated from the skin of frogs. It shares homology with BN at the amidated C-terminal sequence in the final 7 amino acids [33, 34]. GRP is involved in a wide range of physiological functions including exocrine and endocrine secretions, smooth muscle contraction, pain and itch transmission, satiety and behavior [35, 36]. Further, GRP acts as a mitogen, morphogen and pro-angiogenic factor in certain cancers, including PC [37, 38]. Studies show that GRP is higher in PC patients undergoing ADT [27]. Three mammalian receptor subtypes: the neuromedin B-receptor, Gastrin-Releasing Peptide Receptor (GRP-R/BB_2_) and BN receptor subtype 3 have been described for the BN-like family of peptides (reviewed [38, 39]). The GRP-R is the only well characterized receptor to which GRP and BN bind with a high affinity [33, 34]. However, it is unclear about how NED occurs, NE peptides secretion increases in advanced PC, and mechanistically how GRP/GRP-R signaling contributes to progression of CRPC.

In this study, we report that long-term ADT increases the NE hormone GRP and GRP-R expression in PC cells. In addition, constitutive expression of GRP/GRP-R signaling is sufficient to increase ARVs expression through activation of NF-κB signaling resulting in castration-resistant growth of the previously androgen-dependent PC. These findings strongly indicate that the axis of ADT induces GRP/GRP-R activity which activates NF-κB that increases the expression of AR-V7, thereby causing the tumor to progress to CRPC. Further, our studies indicate that GRP-R expression is increased in both of human prostate adenocarcinoma, primary NEPC, and CRPC. Most importantly, GRP/GRP-R signaling is clinically targetable by analogue-based approach, such as GRP-R targeted imaging [40, 41] and radiotherapy [42]. Therefore, GRP/GRP-R may be an important and sufficient target to treat advanced CRPC including AR negative small cell NEPC.

## RESULTS

### Long-term treatment with anti-androgens increases GRP, GRP-R and ARVs expression in PC cells

**Figure 1:**
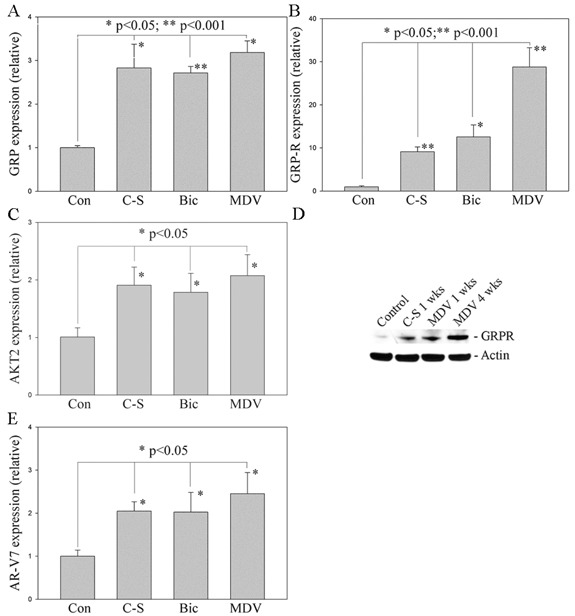
Long-term treatment with anti-androgens induces NED and increases GRP, GRP-R and ARVs expression in PC cells LNCaP cells were treated with media containing charcoal-stripped serum (C-S), Bicalutamide (Bic) or MDV3100 (MDV) for 1-4 weeks. **A.**, **B.** and **C.** GRP, GRP-R and AKT2 expression was determined by qRT-PCR. **D.** GRP-R expression further confirmed by western blot. Actin was used as the loading control. **E.** AR-V7 expression was determined by qRT-PCR. The values plotted represent the mean of at least three individual samples ± SD. Statistical significance was determined by student's *t*-test. * *p* <0.05; ** *p* <0.001.

The recent development of abiraterone acetate, which blocks the synthesis of androgens by the tumor, and the new high affinity anti-androgens, such as enzalutamide (formerly MDV3100), once again blocks AR action in CRPC validating that the AR pathway is a central target for drug therapy [43, 44]. However, in due course, failure of these new drugs occurs. In order to understand the mechanism by which PC escapes from anti-androgen therapy and define a new target to control CRPC progression, we generated resistant PC cells by long-term treatment of PC cells with anti-androgens. We treated androgen dependent LNCaP PC cells with media containing C-S serum (charcoal stripped serum, with no or very low levels androgens), Bicalutamide (Bic) or MDV3100 (MDV) (normal culture media plus Bic/MDV) for 1 to 4 weeks. Our studies show that long-term treatment (4 weeks) with media containing C-S and anti-androgens (Bic or MDV) (which mimics ADT in PC patients) increases expression of GRP, GRP-R and AKT2 (a downstream target gene of GRP/GRP-R signaling) [45, 46] in LNCaP PC cells (Figure 1A, 1B and 1C). GRP-R expression is further confirmed by Western blot assay that show increased expression of GRP-R can be detected as early as one week after treatment with anti-androgens (Figure 1D). In addition, AR-V7 (one of the well-known ARVs) expression also increased significantly in LNCaP PC cells after treatment (4 weeks) with anti-androgens (Figure 1E). These findings suggest that GRP/GRP-R signaling activated after long-term ADT may be involved in anti-androgen resistance. Previously, we have shown that NED and increased NE peptides expression, such as BN and GRP, contribute to castration-resistant growth of PC through activation of NF-κB and AR signaling [14, 15]. Therefore, it is possible that increased GRP expression will affect PC cells by binding to GRP-R through an autocrine and/or paracrine mechanism resulting in progression to CRPC.

### GRP/GRP-R signaling increases ARVs expression through activating NF-κB signaling in PC cells

**Figure 2:**
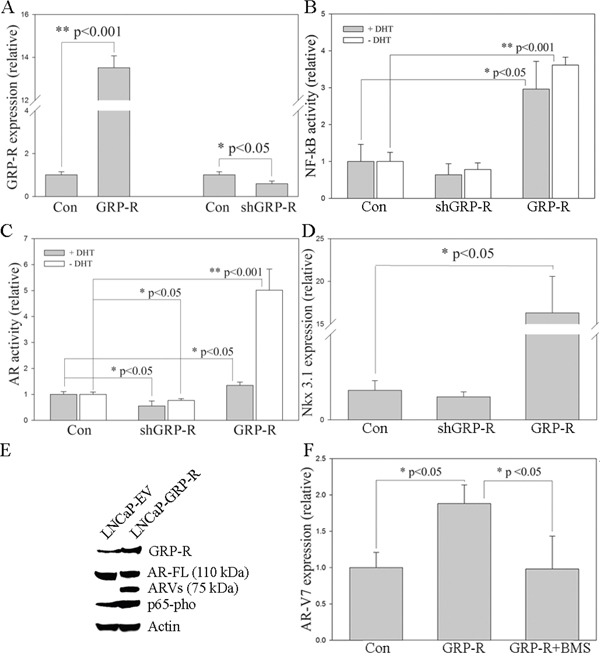
GRP/GRP-R signaling increases ARVs expression through activating NF-κB signaling in PC cells LNCaP cells were infected with GRP-R or shGRP-R expression retroviral vectors. **A.** GRP-R expression was determined by qRT-PCR. **B.** NF-κB activity was determined using NGL, a NF-κB reporter vector. **C.** AR activity was determined using ARR2-PB-Luc, an AR reporter vector. **D.** Nkx 3.1 expression was determined by qRT-PCR. **E.** Full-length AR (AR-FL; N-20 antibody; 110 kDa), ARVs (N-20 antibody; 75 kDa) and pho-p65 expression were confirmed by western blot. Actin was used as the loading control. **F.** AR-V7 expression was determined by qRT-PCR. BMS345541 (BMS; 10^−5^M) was used as the blocker of NF-κB signaling. The values plotted represent the mean of at least three individual samples ± SD. Statistical significance was determined by student's *t*-test. * *p* <0.05; ** *p* <0.001.

In order to understand how GRP/GRP-R signaling supports CRPC progression, we activated or inactivated GRP/GRP-R signaling in PC cells by infecting LNCaP cells with GRP-R or shGRP-R expression viral vectors [47] (Figure 2A). The NGL vector [a NF-κB responsive reporter vector which has Luciferase and Green Fluorescent Protein (GFP) reporter genes] [48] was used to measure NF-κB activity and ARR_2_PB-Luc vector, an AR responsive reporter vector [49], was used to measure AR activity. Our studies show that overexpression of GRP-R increases NF-κB/AR activity and AR target gene (Nkx 3.1) expression (Figure 2B, 2C and 2D), while knock-down of GRP-R inhibited NF-κB and AR activity in PC cells in the presence and absence of androgen (DHT) (Figure 2B and 2C). Most importantly, increased AR activity is greater in the absence than the presence of androgen (5 vs. 1.3 fold increases) (Figure 2C). In addition, over-expression of GRP-R increased ARVs expression (Figure 2E and 2F). These results indicate that GRP/GRP-R signaling activates AR signaling mainly through “ligand-independent manner” by increasing ARVs expression in PC cells. Western blot analysis confirmed that activation of GRP/GRP-R signaling by over-expression of GRP-R increases NF-κB activity (nuclear p65-pho) (Figure 2E).

Recently, we have demonstrated that activation of NF-κB signaling increases ARVs expression in PC cells, thereby promoting progression to CRPC [9]. Therefore, this suggests that activation of GRP/GRP-R signaling followed by long-term ADT contributes to progression of CRPC through activating NF-κB signaling. In order to confirm this hypothesis, BMS345541, a well-known specific inhibitor of the NF-κB pathway that efficiently blocks NF-κB signaling in PC cells [9] was used. As expected, over-expression of GRP-R increased AR-V7 expression in PC cells. However, this elevation was inhibited by blocking NF-κB signaling with BMS345541 (Figure 2F). These results indicate that GRP/GRP-R signaling increases ARVs expression through activating NF-κB signaling in PC cells.

### GRP/GRP-R signaling contributes to progression of PC cells to androgen independent growth by increasing AR-V7 expression

**Figure 3:**
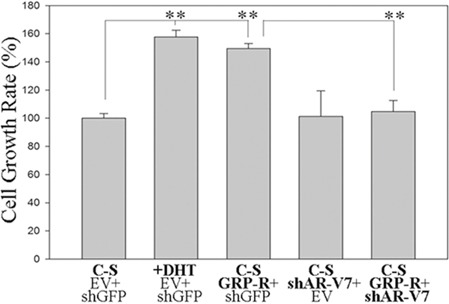
GRP/GRP-R signaling contributes progression of PC cells to androgen independent growth by increasing ARVs expression LNCaP cells were transfected with GRP-R and/or shAR-V7 expression vectors. Then, the cells were treated with (DHT 10^−8^M) or without androgen (media containing charcoal-stripped serum) for 24 hours. The cells transfected with empty retroviral vector and/or PLKO.1 shGFP were used as control shRNA. Cell proliferation assay was performed at 24 hours after transfection. The values plotted represent the mean of at least three individual samples ± SD. Statistical significance was determined by student's *t*-test. ** *p* <0.001.

To test how activation of GRP/GRP-R signaling contributes to androgen independent growth of PC cells, we treated androgen dependent LNCaP cells with C-S media for two days, then transfected the GRP-R expression vector into the LNCaP cells. Cell proliferation assay was performed at 24 hours after transfection. Our results show that over-expression of GRP-R significantly increased cell proliferation in androgen dependent LNCaP cells even in the absence of androgens (Figure 3). Importantly, this effect was inhibited by the knock-down of AR-V7 expression by co-transfection with shAR-V7 expression vector (Figure 3). These results indicate that GRP/GRP-R signaling contributes to progression of PC cells to androgen independent growth by increasing AR-V7 expression

### Activation of GRP/GRP-R signaling contributes to androgen dependent PC tumors to grow in the castrated mice

**Figure 4:**
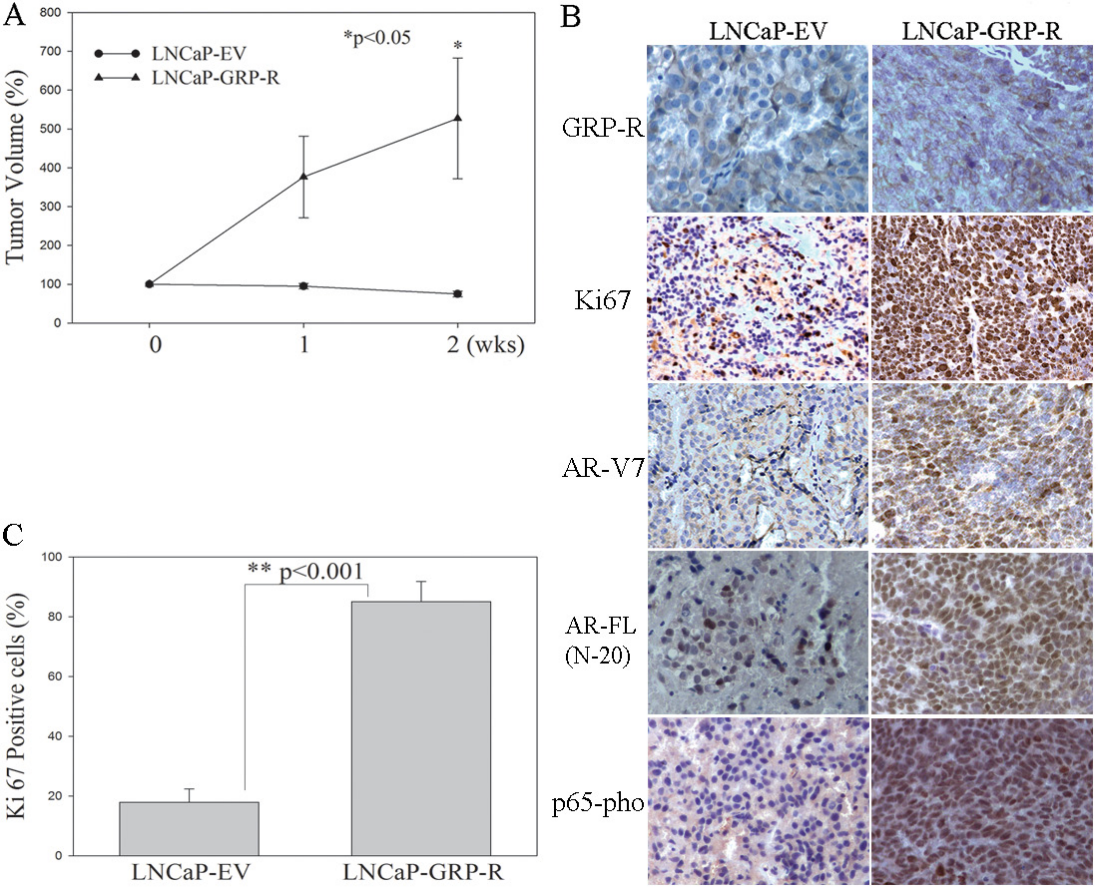
Overexpression of GRP-R increases ARVs expression and contributes to androgen dependent PC tumors to grow in the castrated mice LNCaP-EV and LNCaP-GRP-R cells were injected into flank of male nude mice subcutaneously. After tumor formation (6 weeks), the mice underwent castration. **A.** Tumor volume was measured weekly and the tumors were harvested for the analysis at 2 weeks after castration. **B.** IHC staining was performed to determine GRP-R, Ki67 (proliferation marker), AR-V7, AR-FL (N-20 antibody) and p65-pho expression in the xenografts. **C.** Each tissue section was counted manually in three different areas to assess the Ki67 positive cells index. The data were then presented as number of Ki67 positive cells/100 cells. The results are reported as mean value (%); *bars*, ± SD. * *p* <0.05; ** *p* <0.001 by Student's *t* test (*t* test).

In order to confirm that activation of GRP/GRP-R signaling is sufficient to cause progression to CRPC *in vivo*, we generated GRP/GRP-R signaling activated PC cells by stably infecting androgen dependent LNCaP cells with a GRP-R expression retroviral vector (LNCaP-GRP-R). LNCaP cells are androgen dependent and do not grow in castrated mice. LNCaP cells infected with an empty vector were used as controls (LNCaP-EV). Although overexpression of GRP-R slightly altered proliferation rates, the engineered cells grew well *in vitro* after activation of GRP-R (data not show). We injected the LNCaP-GRP-R or LNCaP-EV cells subcutaneously into the right flank of 7-week-old male athymic nude mice (BALB/c strain). After the primary tumors reached 3-4 mm diameter (6 weeks), all mice were castrated, and the xenograft tissues were harvested two weeks after castration for further analysis. Tumor volume of LNCaP-EV xenografts, as expected, slightly decreased within 2 weeks after castration (Figure 4A). However, GRP/GRP-R signaling activated LNCaP-GRP-R tumors continued to grow after castration (Figure 4A). Immunohistochemical (IHC) staining of Ki67 (proliferation marker), nuclear p65-pho and AR-V7 further confirmed that activation of GRP/GRP-R signaling supports androgen dependent PC tumors to continue to grow in the castrated mice by elevating NF-κB activity and AR-V7 expression in PC cells (Figure 4B and 4C). This strongly supports a mechanism whereby GRP/GRP-R signaling can induce the expression of AR-V7, resulting in progression to CRPC.

### GRP-R expression is elevated in PC patients

**Figure 5:**
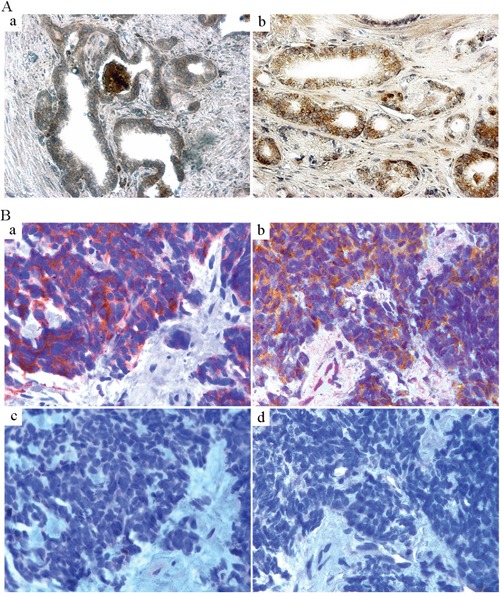
GRP-R expression is increased in human PC **A.** TMA IHC staining of GRP-R was performed to investigate GRP-R expression in human PC. 128 cases of formalin-fixed primary PC tissues with corresponding normal tissues were investigated. a) GRP-R expression in corresponding non-neoplastic prostate tissue. b) GRP-R expression in the PC tissue (71 year old male; Gleason Score 6; T stage 2A). **B.** IHC staining of GRP-R was performed in the human NEPC transrectal biopsy tissues. Seven de-identified pathologically confirmed small cell carcinoma samples (needle biopsy) were investigated. a) IHC staining of Synaptophysin (neuroendocrine marker). b) IHC staining of GRP-R. c) IHC staining of AR (N-20 antibody). d) IHC staining of PSMA.

Based upon our findings, blocking GRP/GRP-R signaling is sufficient to control progression to CRPC. Unfortunately, there are no FDA approved drugs that block GRP/GRP-R signaling. However, GRP/GRP-R signaling is clinically targetable by using analogue-based approach directed against the receptor (GRP-R) [50–52]. In order to determine if GRP-R is expressed and therefore targetable in PC patients, we investigated GRP-R expression in human PC specimens by a Tissue Microarray (TMA) analysis. TMA analysis was performed for GRP-R expression by IHC staining of 128 cases of primary PC patients. Our results show that GRP-R expression is clearly higher in the PC tissues compared to non-neoplastic prostate tissues (Figure 5A). Only one case of PC (0.8%) is negative for GRP-R staining. Among GRP-R positive cases, 110 cases (85.9%) show moderate or high expression of GRP-R (Table 1). These results are consistent with previous reports that GRP-R is expressed at very low levels in normal prostate glands but is increased in human PC [38, 53].

**Table 1:** GRP-R staining (IHC) intensity in 128 cases of formalin-fixed human primary PC tissues

Intensity	(−)	+	++	+++
Gleason's Score				
6 (14 cases)	0	2	5	7
7 (56 cases)	1	5	23	27
8 (10 cases)	0	1	4	5
9 (47 cases)	0	9	16	22
10 (1 cases)	0	0	1	0
**Total** (128 cases)	1 (0.8%)	17 (13.3%)	49 (38.3%)	61 (47.6%)

Published data from clinical trials shows that after failure to abiraterone acetate and enzalutamide, there is an appearance of NEPC in 10-37% of the CRPC patients [13, 21, 25, 54, 55]. It is clear that most NEPC lack AR and NED can result in a loss of AR in the adenocarcinoma (at least some of NED tumors). Circulating tumor cells (CTC) that are NEPC show low or no AR expression (detected by IHC with the amino-terminal that would recognize all forms of the AR [21]); therefore no proof exists if NEPC cells can express the ARVs. However, it is likely that the NEPC undergo an AR-independent transition from adenocarcinoma that no longer needs the AR-signaling pathway to survive and grow. Therefore, inhibiting ARVs expression by blocking GRP/GRP-R signaling may not be sufficient to control AR negative NE tumor growth. However, expression of GRP and GRP-R is an important feature of NED/NE tumor, and GRP-R is clinically targetable by using analogue-based approach directed against the receptor (such as targeted radiotherapy) [50–52]. In order to investigate if GRP-R is targetable in small cell NEPC, we performed IHC staining of GRP-R in a limited set of human NEPC needle biopsy tissues (7 cases). Our studies show that GRP-R expression is clearly high in all of 7 cases of NEPC tissues while AR and prostate specific membrane antigen (PSMA) is negative (Figure 5B).

Together, these results indicate that GRP-R is targetable (such as imaging and/or radiotherapy) for both of AR positive adenocarcinoma and AR negative NEPC.

### GRP-R is amplified in CRPC and therapy induced NEPC

**Figure 6:**
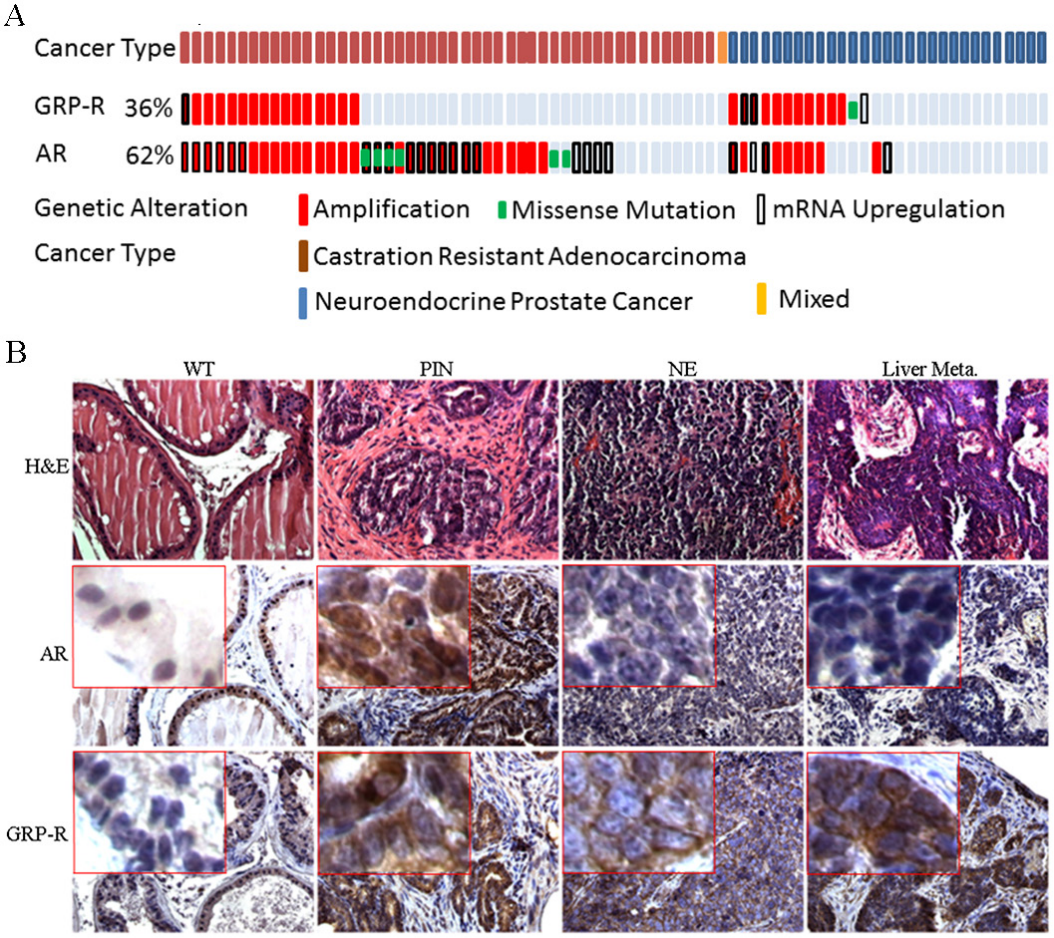
GRP-R is amplified in CRPC and therapy induced NEPC **A.** For genomic analysis of GRP-R expression in CRPC types (adenocarcinoma versus NEPC), data from Beltran *et al* [55], cBioPortal at www.cbioportal.org [56, 57] was utilized. Summary of data generated by cBioPortal was adapted for figure generation. Both AR and GRP-R are amplified in both CRPC adenocarcinoma and NEPC. **B.** IHC staining of AR (N-20 antibody) and GRP-R were performed at the different stages of progression (wild-type, PIN, NEPC and metastatic lesion of the liver) in the TRAMP mice. WT: wild-type; PIN: atypical hyperplasia - mouse PIN lesion; NE: mouse neuroendocrine prostate cancer; Liver Meta: NE metastatic lesions to the liver.

For genomic analysis of GRP-R expression in CRPC (including castration resistant prostate adenocarcinoma and NEPC), data from Beltran *et al* [13], was analyzed using cBioPortal at www.cbioportal.org [56, 57]. Summary of data generated by cBioPortal was adapted for figure generation (Figure 6A). Our results show that the GRP-R gene is amplified and/or over-expressed in 36% of CRPC tumors (28 cases out of 78 cases of CRPC; 33% in adenocarcinoma and 41% in NEPC, respectively) (Figure 6A). In addition, this same dataset demonstrates that 62% of CRPC tumors harbor AR gene amplifications or over-expressions (48 cases out of 78 cases of CRPC) (Figure 6A). Interestingly, 38% of NEPC tumors (11 cases out of 29 cases) showed that AR gene is amplified and/or over-expressed, suggesting that therapy induced NEPC can be both as AR positive and negative (Figure 6A).

In order to determine if GRP-R expression occurs during progression to NEPC in a mouse model, we investigate the GRP-R expression using TRAMP mouse NEPC (mNEPC) model [58]. The TRAMP mouse develops atypical hyperplasia (mouse PIN lesion), papillary tumors, and NE carcinomas in the prostate [58–60]. IHC staining of AR and GRP-R were performed at the different stages of progression in the TRAMP mice. The studies show that GRP-R expression is increased in atypical hyperplasia (mouse prostatic intraepithelial neoplasia PIN lesion) and mouse NEPC, including in the metastatic lesions to the liver; while, AR expression is decreased in PIN lesions and almost undetectable in mNEPC (Figure 6B).

## DISCUSSION

ADT in the majority of PC patients results in initial regression of disease and a dramatic decrease in serum PSA. Eventually, all patients will fail ADT, including developing resistance to the new generation of inhibitors of AR action. Although multiple mechanisms have been proposed to explain escape from ADT (such as AR amplification, modification of the AR by point mutations or phosphorylation, and changes in AR co-activators), recent studies have demonstrated that the elevated expression of ARVs is an important driving force in developing CRPC [1–5]. We have shown that activation of NF-κB signaling increases ARVs (AR-V7) in benign prostatic tissue [8], PC [9], and NF-κB/AR-V7 increases expression of steroid-5α-reductase type II, the enzyme which converts testosterone to dihydrotestosterone, the most active androgen [61]. In support of our reports, Nadiminty et al demonstrated that NF-κB regulates expression of ARVs in CRPC [10]. The detailed mechanism on how NF-κB signaling is activated by ADT has not been reported. Here we show that ADT induces increased expression of GRP and GRP-R resulting in the activation of the NF-κB pathway which induces the expression of ARVs.

NED and neuropeptides secreted from PC cells allow PC cells to adapt to ADT. Previously, we showed that neuropeptides contribute to progression to CRPC through the activation of NF-κB and AR signaling [14, 15]. In this study, we demonstrated that long-term ADT increases neuropeptides (such as GRP) expression in PC cells (Figure 1). This result is consistent with the previous findings that NED is increased within CRPC tumors as well as serum levels of NE-derived peptides such as GRP, neuron-specific enolase, and chromogranin-A are seen in CRPC patients [13, 21, 27–29]. In addition, we demonstrated that activation of GRP/GRP-R signaling increases AR-V7 expression through activation of NF-κB signaling, resulting in progression to CRPC (Figure 2, 3 and 4). Taken together, our findings strongly indicate that ADT induces GRP and GRP-R expression in PC cells. Increased GRP will bind to GRP-R through autocrine and/or paracrine signaling to activate GRP/GRP-R signaling. Activated GRP/GRP-R signaling increases ARVs expression through NF-κB signaling; thereby, contributing to progression to CRPC. This is an important mechanism whereby tumors can escape hormonal therapy and the GRP-R provides a new therapeutic target to treat hormonal resistant PC.

NEPC is generally reported to lack AR and NED results in a decrease/loss of AR in the adenocarcinoma. However, transdifferentiation of the adenocarcinoma to NEPC appears to occur in stages where tumors with NE features may still have AR or express AR target genes. In 95 primary prostate small cell carcinomas, 25% were positive, often only focally, for prostate specific membrane antigen (PSMA) while 28% showed detectable PSA [32]. A new study shows that 10.7% of CRPC patients have CTCs that are NEPC cells which have either low or undetectable levels of AR [21]. Using a mouse model of NEPC (LPB-Tag transgenic line12T-10), we reported that PIN, a preneoplastic lesion for PC, shows strong staining for both chromogranin A (a NE-marker) and AR. However, progression in the mouse to classic small cell carcinoma pathology is accompanied by weak to negative staining for the AR [62]. Thus, both human and mouse models show AR positive cells during progression to NEPC but likely the final stage results in an AR negative PC that no longer expresses androgen target genes and fails to respond to hormonal therapy (see review [22]). There are no studies that report the existence ARVs in NED or NEPC. However, classic AR negative NE/NED tumors would have no need for ARVs expression to survive and grow.

Regardless of AR status, it is important to note that NE cells produce mitogens (e.g. insulin-like growth factor I [IGF-I] or GRP) that act in a paracrine or autocrine manner to activate their cognate receptors and stimulate proliferation [63]. These NE secretions can impact on the growth of the adenocarcinoma. For example, we reported that a NE mouse PC would cause an LNCaP xenograft to grow in castrated mice [14]. Therefore, ADT will not eliminate the AR negative NE-like cells rather it will increase progression to these NE tumors. Several studies have shown that GRP/GRP-R targeted therapy inhibits neuroblastoma tumor growth and both AR positive and negative PC tumors [38, 39]. These findings suggest that GRP/GRP-R can provide support for cancer cell survival and proliferation. Consistent with published studies [38, 53], we demonstrated that GRP-R expression occurs in prostate adenocarcinomas and is increased in human small cell NEPC tumors (Figure 5). In addition, our analysis of published genomic sequencing of advanced PC patients revealed that the GRP-R gene is amplified in 41% of NEPC tumors resulting in overexpression of the GRP-R mRNA. This same dataset demonstrates that NEPC appear both as AR positive and negative (Figure 6A). Also, we show that GRP-R expression increases during progression of the mouse TRAMP tumor from PIN to NEPC (Figure 6B). Therefore, we propose that GRP-R can be a direct target of intervention to block progression or treat CRPC, including AR negative NEPC.

New imaging methods have been developed that follow metastatic PC by targeting the PSMA receptor [64]. However, one imaging study showed that a NEPC did not express PSMA and therefore cannot be detected by PSMA imaging [65]. Using GRP-R targeted Ga^68^-BAY86-7548, a BN-analogue [52], it is possible to follow expression of GRP-R in both the AR positive adenocarcinomas as well as the AR negative NEPC tumors. A newly published imaging study confirms that GRP-R targeted PET/CT clinical imaging, using a radiolabeled BN-analogue, successfully detected primary, recurrent and metastatic lesions of PC, and displayed good tumor delineation in a subset of patients with recurrent PC, including lymph node and bone metastatic lesions in patients with PC [52]. These results confirm that GRP-R is overexpressed in primary, recurrent and metastatic lesions of PC patients at sufficiently high levels to allow for selective and effective targeting of the tumor. Using both PSMA and GRP-R imaging in the same patient [41], it would be possible to detect both the androgen regulated PSMA expression adenocarcinomas as well as progression to the PSMA/AR negative NEPC. Further, this BN-analogue can be developed for radiotherapy to kill NEPC as well as any GRP-R positive tumors.

**Figure 7:**
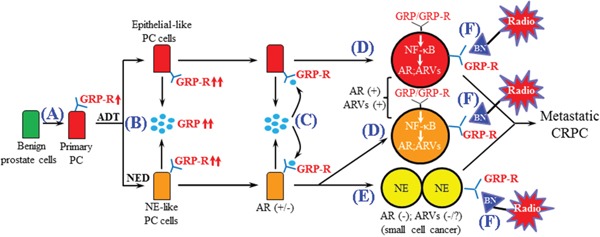
Schematic representation of the role of GRP/GRP-R signaling in the progression of CRPC **A.** The GRP-R expression is increased in PC patient. **B.** ADT induces NED and further increases GRP and GRP-R expression. **C.** GRP secreted from PC cells affects PC cells by binding to GRP-R through autocrine and/or paracrine signaling. **D.** Activated GRP/GRP-R signaling increases ARVs expression through NF-κB signaling thereby contributing to progression to CRPC (including NE-like PC). **E.** Once PC progresses to NEPC, PC cells will lose AR, becoming resistant to anti-androgen treatment. However, GRP-R positive NE cancer growth is stimulated by BN/GRP. Therefore, F. Both AR (+) and AR (−) CRPC will be sensitive to the GRP-R targeted imaging and radiotherapy.

In summary, the results strongly indicate that the axis of ADT induces GRP/GRP-R activity, activation NF-κB and increased expression of ARVs. With expression of ARVs, we have reached the first stage of failure to drugs that block the LBD of the AR-FL. New drugs are now under development that will block the activity of the ARVs [66, 67]. However, transdifferentiation of the adenocarcinoma to NEPC presents a new challenge to develop an approach to target the AR negative stage of CRPC. We have developed a working model that integrates our findings with the progression to CRPC (Figure 7). (A) The GRP-R expression is increased in PC patients. (B) In PC patients, ADT will induce NED/NE transdifferentiation (to create mosaic tumors with NED and NEPC) that results in an increase of the NE hormone GRP and the GRP-R expression. (C) NE peptides secretion (BN/GRP) from PC cells and NE-like cells, bind to GRP-R through autocrine and/or paracrine signaling. (D) Activated GRP/GRP-R signaling will increase ARVs expression through NF-κB signaling, and this enhanced expression of ARVs, which lack the LBD, will contribute to PC cells (including AR positive NE-like PC cells) to become resistant to anti-androgen treatment. (E) Once PC progresses to NE cancer, PC cells will lose AR, becoming resistant to any drug that targets the AR. However, GRP-R positive NE cancer growth is stimulated by BN/GRP. Therefore, (F) AR positive CRPC and AR negative NEPC will be respond to the GRP-R targeted imaging and radiotherapy.

## MATERIALS AND METHODS

### Cell culture and materials

The human prostate carcinoma cell line LNCaP was obtained in 2012 from the ATCC (Manassas, VA). Cells were maintained at 37°C in a humidified atmosphere of 5% CO_2_ in the air, and were tested for contamination within the past 6 months using a Mycoplasma Detection Kit (SouthernBiotech). Cell line was routinely cultured in RPMI 1640 (Gibco-BRL) medium containing 5% fetal calf serum (FBS) (Hyclone), 0.1% ITS and 0.1% Glutamine (Gibco-BRL). The following reagents were purchased for *in vitro* experiments: BMS345541 (Sigma-Aldrich), Bicalutamide (Selleckchem) and MDV3100 (Sigma).

### Reverse transcription and real-time PCR

Total RNAs from experimental cells were extracted using Trizol (Gibco-BRL), and residual genomic DNA was removed by DNaseI (Invitrogen) treatment. The RNAs were reverse transcribed using random primers and Superscript II (Gibco-BRL) according to the manufacturer's protocol. The primers used to amplify GRP were 5′-GCTGGGTCTCATAGAAGCAAAG-3′ (forward), 5′-TGGAGCAGAGAGTCTACCAAC-3′ (reverse); primers of GRP-R were 5′-GCTGGCCATTCCAGAGGCCG-3′ (forward), 5′-CGACAGTGGGATGACGTAGAAGACCA -3′ (reverse); primers of AR-V7 were 5′-CCATCTTGTCGTCTTCGGAAATGTTATGAAGC-3′ (forward), 5′- TTTGAATGAGGCAAGTCAGCCTTTCT-3′ (reverse) [2]. Real-time PCR reactions were carried out in a 20μl volume using a 96-well plate format and fluorescence was detected utilizing the Bio-Rad I-Cycler IQ Real-time detection system. Gene expression was normalized to 18s rRNA by the 2^−ΔΔCt^ method [68]. The values plotted represent the mean of at least three individual samples ± SD.

### Western blot analysis

Whole cell lysate was extracted from experimental cells. A 20μg aliquot of each protein sample was separated on a 4 to 12% Tris-glycine gradient gel (NOVEX^TM^), and then transferred to nitrocellulose membranes (Schleicher & Schuell, Germany). The membranes were blocked with 5% skim milk in TBS-T (Trypsin buffered saline, 1% Tween-20) buffer. The AR (N-20, Santa Cruz; 1:1000), p65-pho (Abcam; 1:300) or GRP-R (Abcam; 1:200) antibody was added and the blots were incubated o/n in 4 C°. After washing three times for 10 minutes each in TBS-T, incubation was performed for 1 hour with the secondary horseradish-peroxidase-conjugated anti-rabbit/anti-mouse antibody. Actin was used as the loading control. The signals were developed by an ECL detection system (Amersham Biosciences, Amersham, USA).

### Generation of GRP/GRP-R signaling activated/inactivated PCa cells

To generate GRP/GRP-R signaling activated/inactivated PCa cells, LNCaP cells were infected with GRP-R expression retroviral vector (LNCaP-GRP-R) [47]. The cells infected with empty vector were used as controls (LNCaP-EV).

### Transient transfection assay

The NGL vector [a NF-κB responsive reporter vector which has Luciferase and Green Fluorescent Protein (GFP) reporter genes] [48] was used to measure NF-κB activity and the ARR_2_PB-Luc vector (an AR responsive reporter vector) [49] was used to measure AR activity in the PC cells by transient transfection experiments. LNCaP cells were plated at an initial density of 2.5 × 10^4^/well in 24-well tissue culture plates. After 24 hours, the cells were transfected with NGL/ARR_2_PB-Luc vectors using Lipofectamine (Invitrogen) for four hours according to the manufacturer's protocol. Luciferase activity was determined using the Promega Corp luciferase assay system 24 hours after transfection. The transfection efficiency was determined by co-transfecting pRL-CMV containing the Renilla luciferase reporter gene (Promega). The values plotted represent the mean of at least three individual samples ± SD.

### Proliferation assay

Experimental cells (LNCaP) were treated with media containing charcoal-stripped (C-S) serum (no or very low levels androgens) for 24 hours. Then, cells were plated in a 96-well plate (2 × 10^4^/well). After 24 h, the cells were transfected with GRP-R expression retroviral vector and/or shAR-V7 expression vector (PLKO.1 shAR-V7; gift from Dr. Kerry L. Burnstein at University of Miami) using Lipofectamine (Invitrogen) for four hours according to the manufacturer's protocol. The cells transfected with empty retroviral vector (EV) and/or PLKO.1 shGFP were used as controls. Cell proliferation assay was performed at 24 hours after transfection. All of the measurements were carried out in triplicate.

### CRPC xenograft mouse model

All animal studies were carried out in strict accordance with the recommendations in the Guide for the Care and Use of Laboratory Animals of the National Institutes of Health. The protocol was approved by the Vanderbilt Institutional Animal Care & Use Committee (Permit Number: M/09/387). CRPC xenograft mouse model was generated by injection of GRP/GPR-R activated LNCaP (LNCaP-GRP-R) cells subcutaneously into the right flank of 7-week-old male athymic nude mice (BALB/c strain). After the primary tumors reached 3-4 mm diameter (6 weeks), the mice were castrated. Control group mice were injected with LNCaP cells infected with control empty vehicle vector (LNCaP-EV). Tumor volume was measured weekly and calculated by the formula: Volume = π/6 × W × H × L (mm^3^). At 2 weeks after castration, the xenograft tissues were harvested and fixed in 10% buffered formalin and paraffin embedded for histologic and immunohistochemical analyses. Each group had at least five mice. The results are reported as the mean percent ± SD.

### Immunohistochemistry

Paraffin-embedded tissue sections were stained immunohistochemically with antibodies against GRP-R (Abcam), Ki67 (clone TEC-3, DAKO), AR (N-20, Santa Cruz), p65-pho (Abcam) and AR-V7 (Precision). The primary antibody was incubated at the appropriate concentration (GRP-R, 1:200; Ki67, 1:1000; AR, 1:1000; p65-pho, 1:1000; AR-V7, 1:200) for one hour at room temperature. The secondary antibody was incubated for 60 minutes. Slides were rinsed extensively in tap water, counterstained with Mayer's hematoxylin and mounted.

### Human prostate cancer samples

Tissue microarray used for immunohistochemical staining (IHC) of GRP-R was purchased from Accurate Chemical and Scientific Corporation (Westbury, NY). AccuMax^TM^ arrays (A222III, A223II, A302IV and A718VII) contain total 128 cases of formalin-fixed primary PC tissues with corresponding normal tissues. The specimens are from radical prostatectomy. Seven de-identified pathologically confirmed small cell carcinoma samples, obtained through transrectal biopsy, were obtained through collaboration with O.U.R. Labs (Nashville, TN) in 2004, as previously reported [69]. Sample use was approved through the Institutional Review Board. Expression across the TMA and needle biopsies was scored for intensity across the tissue by a board-certified pathologist (JMC).

### Statistical analysis

Where appropriate, experimental groups were compared using two-sample *t-*test, with significance defined as *p* <0.05.
